# miR-29b-3p regulated osteoblast differentiation via regulating IGF-1 secretion of mechanically stimulated osteocytes

**DOI:** 10.1186/s11658-019-0136-2

**Published:** 2019-03-14

**Authors:** Qiangcheng Zeng, Yang Wang, Jie Gao, Zhixiong Yan, Zhenghua Li, Xianqiong Zou, Yanan Li, Jiahui Wang, Yong Guo

**Affiliations:** 1 key laboratory of Functional Bioresource Utilization in University of Shandong, Shandong Key Laboratory of Biophysics, Dezhou University, Dezhou, 253023 China; 2 Department of Biomedical Engineering, College of Biotechnology, Guilin Medical University, No. 1 Zhiyuan Road, Lingui District, Guilin City, 541100 Guangxi China; 3 Key Laboratory for Biorheological Science and Technology of Ministry of Education, State and Local Joint Engineering Laboratory for Vascular Implants, Bioengineering College of Chongqing University, Chongqing, 400044 China; 4 Medical Department, Secondary Renmin Hospital of Dezhou, Dezhou, 253023 Shangdong China

**Keywords:** Mechanical tensile strain, Osteocyte, Osteoblast differentiation, miRNA microarray

## Abstract

**Background:**

Mechanical loading is an essential factor for bone formation. A previous study indicated that mechanical tensile strain of 2500 microstrain (με) at 0.5 Hz for 8 h promoted osteogenesis and corresponding mechanoresponsive microRNAs (miRs) were identified in osteoblasts. However, in osteocytes, it has not been identified which miRs respond to the mechanical strain, and it is not fully understood how the mechanoresponsive miRs regulate osteoblast differentiation.

**Methods:**

Mouse MLO-Y4 osteocytes were applied to the same mechanical tensile strain in vitro. Using molecular and biochemical methods, IGF-1 (insulin-like growth factor-1), PGE2 (prostaglandin E2), OPG (osteoprotegerin) and NOS (nitric oxide synthase) activities of the cells were assayed. MiR microarray and reverse transcription-quantitative polymerase chain reaction (RT-qPCR) assays were applied to select and validate differentially expressed miRs, and the target genes of these miRs were then predicted. MC3T3-E1 osteoblasts were stimulated by the mechanical tensile strain, and the miR-29b-3p expression was detected with miR microarray and RT-qPCR. Additionally, the effect of miR-29b-3p on IFG-1 secretion of osteocytes and the influence of conditioned medium of osteocytes transfected with miR-29b-3p on osteoblast differentiation were investigated.

**Results:**

The mechanical strain increased secretions of IGF-1 and PGE2, elevated OPG expression and NOS activities, and resulted in altered expression of the ten miRs, and possible target genes for these differentially expressed miRs were revealed through bioinformatics. Among the ten miRs, miR-29b-3p were down-regulated, and miR-29b-3p overexpression decreased the IGF-1 secretion of osteocytes. The mechanical strain did not change expression of osteoblasts’ miR-29b-3p. In addition, the conditioned medium of mechanically strained osteocytes promoted osteoblast differentiation, and the conditioned medium of osteocytes transfected with miR-29b-3p mimic inhibited osteoblast differentiation.

**Conclusions:**

In osteocytes (but not osteoblasts), miR-29b-3p was responsive to the mechanical tensile strain and regulated osteoblast differentiation via regulating IGF-1 secretion of mechanically strained osteocytes.

## Introduction

Mechanical stimulation plays an essential role in the metabolic balance of bone. Physiological loading can induce bone formation, whereas a lack of loading or excessive loading leads to bone resorption [1–4].

As the dominant cells in bone tissue, osteocytes respond to mechanical stimulation, sense and integrate mechanical stimuli into biochemical signals to regulate both bone formation and resorption [5]. Previous studies mainly focused on osteocytes’ response to fluid shear stress which inhibits osteocytes apoptosis and promotes survival by modulating the Bcl-2/Bax expression ratio, enhances expression levels of NO and PGE2, and increases COX2 and the OPG/RANKL ratio, playing a dominant role in regulating the activities of both osteoblasts and osteoclasts [6–8], thus regulating bone reconstruction and remodeling. However, how osteocytes convert the mechanical stimulation into a biological signal and regulate bone formation (activity of osteoblasts) or resorption (activity of osteoclasts) remains not fully elucidated.

MiRs are small non-coding, single-strand RNAs, which control gene expression by targeting to 3′ untranslated regions of mRNA resulting in translational repression or degradation [9]. It was previously found that miR plays a pivotal role in bone formation [10], and many miRs which regulate bone formation have been identified [10, 11]. Some mechanoresponsive miRs were recently identified, they played significant roles in bone formation. For example, miR-33-5p and miR-132 are responsive to mechanical loading and regulate osteogenesis via targeting Hmga2 and mTOR signaling pathway, respectively [12, 13]. Our previous study confirmed that a mechanical tensile strain of 2500 με at 0.5 Hz for 8 h promoted osteogenesis and mechanoresponsive miRs in osteoblasts were identified [14]. The study urged us to investigate osteocytes’ response to the mechanical tensile strain and to search for mechanoresponsive miRs of osteocytes.

miR-29b regulated osteoblast differentiation (in MC3T3 osteoblasts, miR-29b overexpression promotes osteogenic differentiation) [15], and IGF-1 was confirmed to be a target gene of miR-29b [16, 17]. We speculated that miR-29b was responsive to mechanical strain applied to osteocytes and involved in osteoblast differentiation. However, the mechanism by miR-29b osteocytes convert a mechanical signal into a biological signal and regulate osteoblast differentiation has not been fully elucidated.

In this study, the osteocytes’ biological response to a mechanical tensile strain of 2500 με at 0.5 Hz for 8 h was investigated, and some novel mechanosensitive miRs were selected. In addition, the involvement of miR-29b in osteocytes’ response to mechanical strain and osteoblast differentiation were studied.

## Methods

### Cell culture

A mouse MLO-Y4 osteocyte cell line (provided by JENNIO Biological Technology, Guangzhou, China) was cultured in dishes with α-MEM medium (α-MEM, Invitrogen), containing 10% FBS and 1% penicillin. Then the cells were transferred to mechanical loading dishes that were reformed from cell culture dishes (Nalge Nunc International).

Mouse MC3T3-E1 osteoblastic cells (JENNIO Biological Technology, Guangzhou) were cultured with the same medium as mentioned above.

### Application of mechanical strain

At confluence, the medium was renewed with FBS-free medium, then the MLO-Y4 cells were stimulated with mechanical tensile strain of 2500 με at 0.5 Hz for 8 h by a four-point bending device, as previously described [18].

### Enzyme-linked immunosorbent assay (ELISA)

Following mechanical tensile strain, the expression levels of IGF-1 and PGE2 in the collected culture supernatant were detected using an IGF-1 ELISA kit (Boster Bioengineering Co., Ltd., Wuhan China) and PGE2 EIA kit (Cayman Chemical, Michigan USA), according to the manufacturers’ instructions. An ELISA reader (Thermo Scientific Multiskan FC ELISA Reader, Rockford, IL, USA) was used to measure the absorbances at 450 nm and 420 nm respectively, with the results presented as the content of changes, compared to the unstrained control.

### Western blot

Following mechanical tensile strain, cell lysates were prepared in RIPA lysis buffer (Beijing Solarbio Science & Technology, Co. Ltd., Beijing, China). Protein in cell lysates was quantified using the BCA method. Equal amounts of proteins were separated by electrophoresis on a polyacrylamide gel containing 0.15% SDS, then transferred onto PVDF membranes (Millipore, Bedford, MA, USA). After blocking with 5% skim milk and incubation with primary antibodies overnight at 4 °C, the membranes were incubated with horseradish peroxidase conjugated secondary antibody. The immunoreactive bands were visualized using an ECL detection kit (7 sea biotech Co. Ltd., Shanghai, China). β-actin in cell lysates was used as a loading control. Data were normalized against those of corresponding optical density of β-actin.

### Detection of NOS activity

After mechanical tensile strain of MLO-Y4 for 8 h, cells were collected and bathed gently in an ultrasonic processor (UP 400S, Hielscher, Germany) for 2 min. NOS activity was measured using a colorimetric method based on NOS ability to catalyze L-Arg and molecular oxygen to generate NO, and generated NO produces colored compounds with nucleophiles. The optical densities at 530 nm wavelength were obtained using an ELISA reader (Thermo Scientific Multiskan FC ELISA Reader) and activities of NOS were calculated according to the calibration formula provided in the instructions.

### Microarray and RT-qPCR validation of miR

The RiboArray miDETECT mouse array (Ribobio Co., Guangzhou, China) and RT-qPCR were used to detect and validate the miR expression levels in MLO-Y4 cells. The miR expression levels of the mechanically strained group were compared with the unstrained group.

Briefly, total RNA extraction and miR enrichment procedures were performed using the Trizol method and an mirVana miR Isolation kit (Ambion Life Technologies, Carlsbad, CA, USA), according to the manufacturer’s instructions. Target labeling, hybridization, imaging and data processing were performed, according to the manufacturer’s instructions using a RiboArray miDETECT mouse array (Ribobio Corporation) which contained all mouse miRNAs of Sanger miRBase 19. Data were acquired using Agilent Feature Extraction software version 10.7 (Agilent, Palo Alto, CA, USA) [16, 17]. Further data analyses were performed using GeneSpring GX 10.0 software (Agilent, Palo Alto, CA, USA). Following microarray detection, expression levels of 40 miRs with significant differences were validated by RT-qPCR at Ribobio Co., Ltd. in Guangzhou. The miDETECT A Track Uni-Reverse Primers and miDETECT A Track miRNA Forward Primers (specific primers) for RT-qPCR of these miRs were provided by Ribobio Corporation (Ribobio Co., Ltd. Guangzhou, China). Poly(A) tailing, reverse transcription and qPCR were performed successively using the miDETECT A Track miRNA qRT-PCR Starter Kit (Ribobio Co., Ltd). The reactions were incubated in a 96-well optical plate at 95 °C for 20 s, followed by 40 cycles of 10 s at 95 °C, 20 s at 60 °C, and 10 s at 70 °C. Expression analysis was performed in triplicate for each sample. U6 was used as the normalization control. The miR expression levels were quantified using a CFX 96 system (Bio-Rad Laboratories, Hercules, CA).

### miRs transfection and preparation of osteocytes’ conditioned medium

MLO-Y4 osteocyte cells, at 70% confluence, were transfected by miR-29b-3p mimic, miR-29b-3p inhibitor and miR control (Ribobio Co., at a final concentration of 50 nM) respectively, using the riboFect CP Transfection Kit (Ribobio Co.) according to the manufacturer’s method.

The cells were carefully washed in serum-free medium to remove proteins from the bovine serum supplement and then incubated in fresh serum-free medium for 24 h (stimulated with mechanical tensile strain or not). After centrifugation at 3000 g for 12 min, the conditioned medium was collected and prepared for the next experiment.

### Detection of osteoblastic differentiation

MC3T3-E1 osteoblastic cells at confluence were carefully washed in serum-free medium, then the cells were cultured in osteocytes’ conditioned medium for 24 h. Cells were harvested, washed with a phosphate buffer solution (PBS), and lysed with a lysis buffer (Beijing Solarbio Science & Technology, Co. Ltd). The ALP activity of the lysates was measured with the ALP Activity Assay Kit (Nanjing Jiancheng Biotechnology Co. Ltd., China) at 25 °C according to the provider’s protocol. The bone morphogenetic protein 2 (BMP-2) of the osteoblastic cells was assayed with an ELISA kit (Elabscience Biotechnology Co., Ltd., Wuhan, China), according to the manufacturer’s instructions mentioned above.

cDNA was synthesized using the TIANScript RT kit (Tiangen Biotech Co., Ltd., Beijing, China), then the Runx 2 mRNA was detected using SYBR Green I PCR Mix (Beijing CoWin Biotech Co., Ltd., Beijing, China) according to the manufacturer’s method. The PCR amplification reaction included a denaturation step at 94 °C for 3 min followed by 40 cycles of 94 °C for 15 s, annealing at 60 °C for 30 s, and extension at 72 °C for 30 s. Relative expression was normalized to mRNA levels of GAPDH using the 2^-ΔΔ^Cq method.

### Bioinformatics analysis

miRWalk2.0 (http://zmf.umm.uni-heidelberg.de/apps/zmf/mirwalk2/), MicroRNA.

org (www.microrna.org/) and TargetScan (www.targetscan.org/) were applied to predict target genes for these differently expressed miRs. The same target genes for one corresponding miR presented in three online databases were considered as potential targets.

### Statistical analysis

All data are presented as the mean ± standard deviation from three separate experiments (*n* = 5 or 6). Data were tested for normal distribution using the Shapiro-Wilk test and differences between groups were analyzed using one-way analysis of variance and determined by the least significant difference test. Statistical analysis was performed using SPSS software (version 18; SPSS, Inc., Chicago, IL, USA). *P* < 0.05 was considered to indicate a statistically significant difference.

## Results

**Fig. 1:**
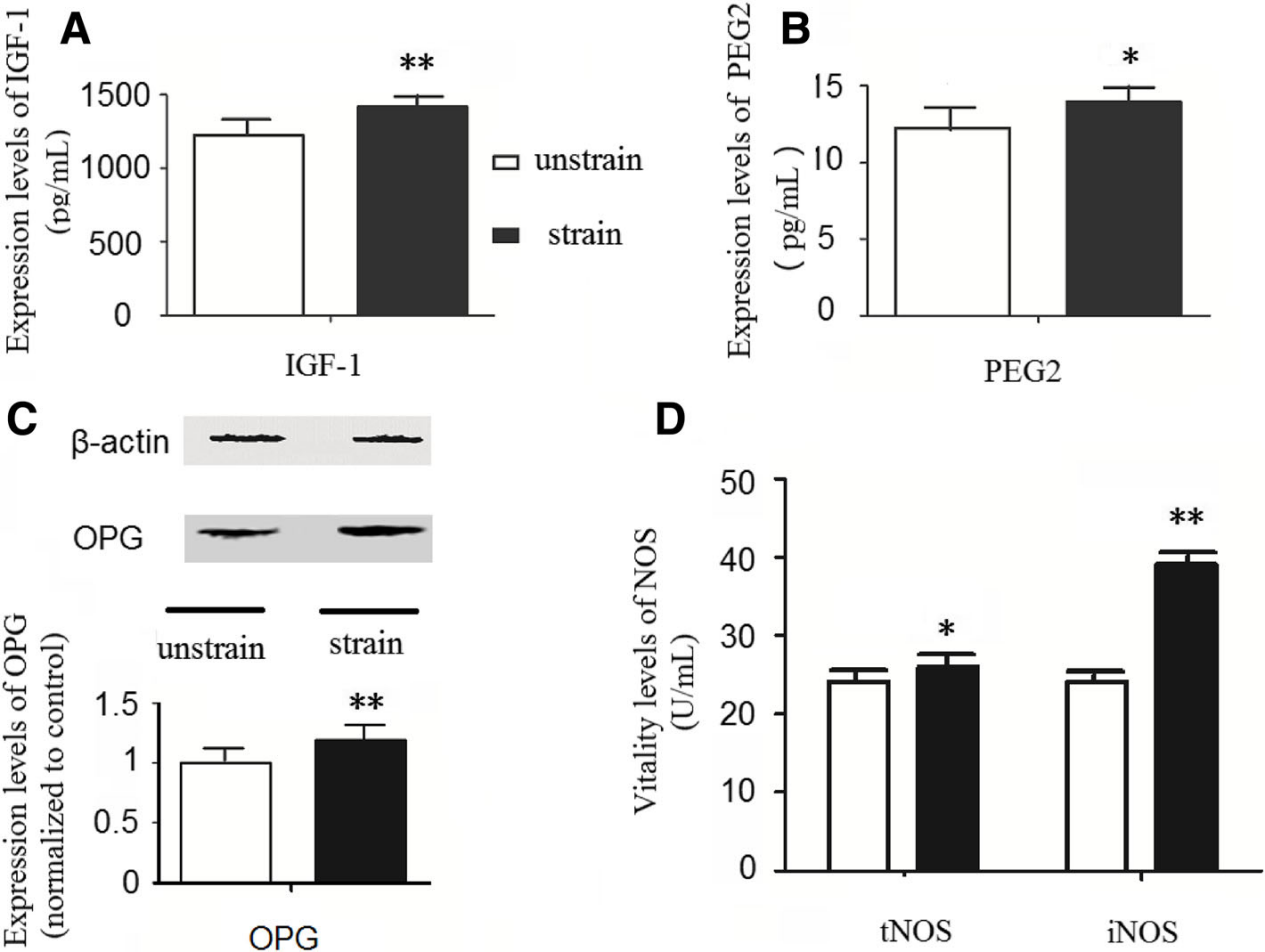
MLO-Y4 cells responded to a mechanical tensile strain of 2500 microstrain (με) at 0.5 Hz for 8 h. **a/b** After MLO-Y4 cells were stimulated by the mechanical tensile strain, the contents of IGF-1 and PGE2 in the supernatant of cell culture medium were increased (*n* = 5). **c** The mechanical tensile strain increased protein levels of OPG (osteoprotegerin) of MLO-Y4 cells (*n* = 6). **d** Following mechanical tensile strain, the activities of NOS in the cells were up-regulated (*n* = 5). *P < 0.05 and **P < 0.01, compared with the unstrained control group

We first investigated osteocytes’ response to a physiological mechanical tensile strain of 2500 με at 0.5 Hz for 8 h. As shown in Fig. 1, the results of ELISA for IGF-1 and PGE2, western blot for OPG and biochemical method for NOS activity were all changed after applying the mechanical tensile strain to MLO-Y4 cells. All four factors play important roles in bone formation: IGF-1 is widely distributed and plays a significant role in bone development via endocrine, paracrine and autocrine by combining with its ligands [23, 24], PGE2 is a necessary factor in gap junction mediated intercellular communication in osteocytes [25]. OPG has been reported to promote bone formation and its absence caused onset of osteoporosis and arterial calcification in mice [26], and NOS is closely associated with the expression level of NO, which could regulate the activity of osteoblasts [27].

**Fig. 2:**
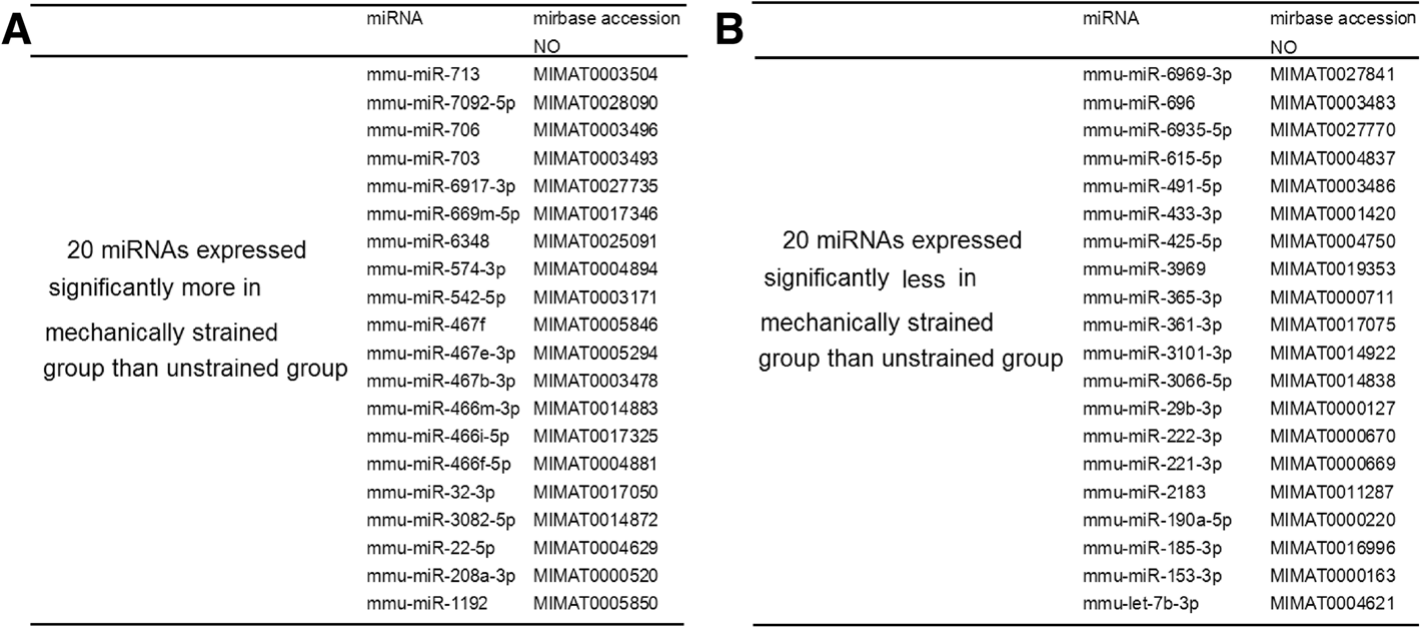
A total of forty differentially expressed miRNAs were selected by microarrays. **a**: up-regulated miRs, **b**: down-regulated miRs

**Fig. 3:**
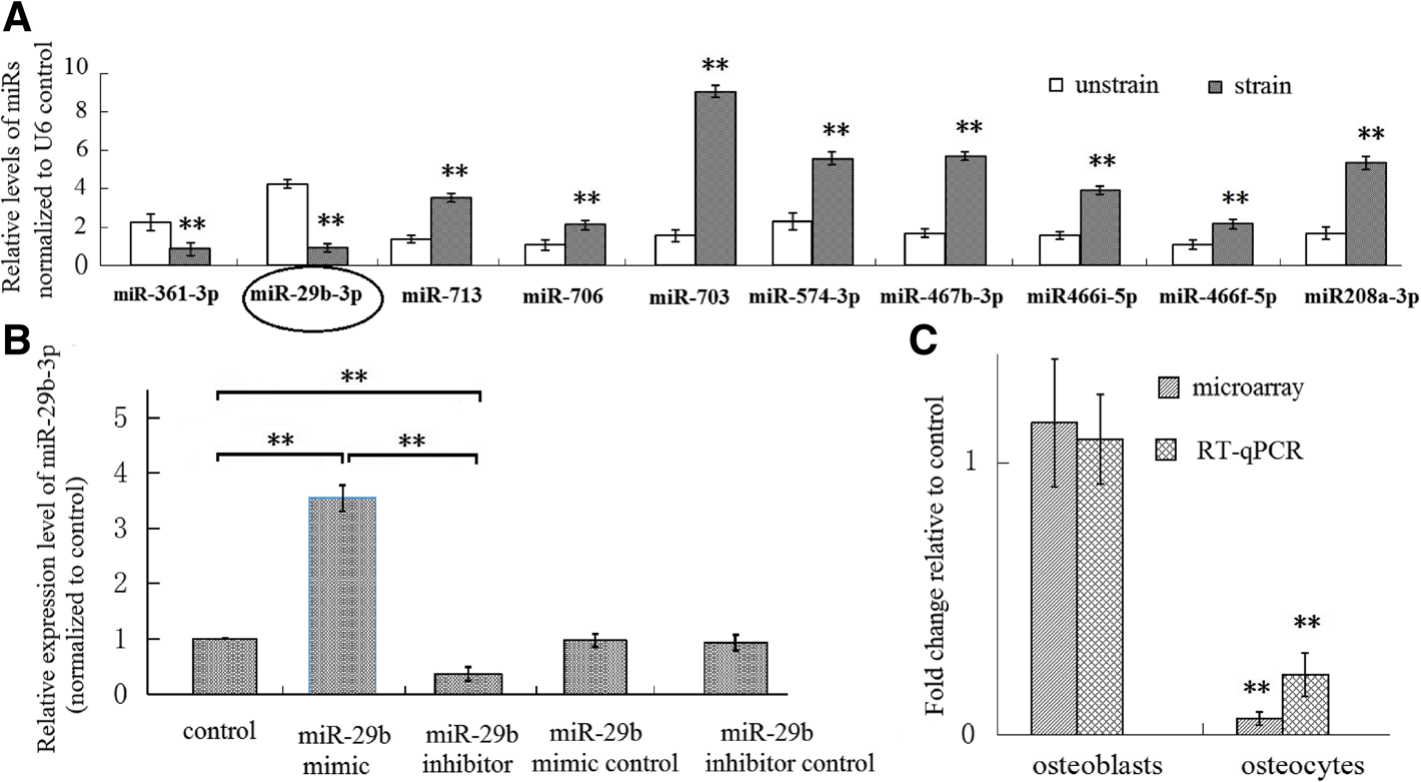
In MLO-Y4 osteocytes, miR-29b-3p was one of the ten mechanoresponsive miRs which were confirmed by RT-qPCR. **a** Based on microarray assay of miRs, the result of RT-qPCR showed that ten miRs were responsive to the mechanical tensile strain, and miR-29b-3p was one of the ten miRs (*n* = 5). **b** The miR-29b-3p mimic elevated miR-29b-3p level of osteocyte and the miR -29b-3p inhibitor decreased the miR level (*n* = 5). **c** In MC3T3-E1 osteoblastic cells, the mechanical tensile strain did not change the expression of miR-29b-3p, which was demonstrated with microarray and RT-qPCR. Contrarily, in MLO-Y4 osteocytes, the mechanical strain decreased miR-29b-3p expression (*n* = 5). *P < 0.05 and **P < 0.01, compared with the unstrained control group, or between the indicated groups

Next, we screened and validated 10 miRs’ response to the mechanical tensile strain applied to MLO-Y4 cells. A total of 40 miRs were differentially expressed through a miR microarray screen (Fig. 2). Then, using qPCR, the 40 miRs were validated further. The results of miR microarray and qRT-PCR indentified 10 miRs that were mechanoresponsive miRs: miR-713, miR-706, miR-703, miR-574-3p, miR-467b-3p, miR-466i-5p, miR-466f-5p and miR -208a-3p were up-regulated and miR-29b-3p and miR-361-3p were down-regulated, compared to the unstrained group (Fig. 2, Fig. 3a).

Bioinformatics was applied to predict target genes for these differently expressed miRs. The results indicated that several target genes were in correlation with bone formation (Table 1). In all of the putative target genes, we found 29 genes that correlated with bone formation and might play important roles in bone formation. Of all the target genes, IGF-1 attracted our attention the most: IGF-1 was a target gene of miR-29b-3p, confirmed by previous studies [16, 17]. In our study, the expression level of IGF-1 was increased (Fig. 1) while the expression level of miR-29b-3p was decreased in mechanically strained osteocytes (Fig. 3a), indicating that miR-29b-3p was likely to regulate bone formation via targeting IGF-1.

**Table 1:** Predicted target genes of the miRs

miRNA	Annotation and Reference	RefseqID
mmu-miR-29b-3p	IGF-1, Insulin-like Growth Factor-1[23, 24]	NM_000076.6
	Grip 1, Glutamate Receptor-Interacting Protein 1 [28]	NM_028736
	Dtx4, deltex 4, E3 ubiquitin ligase [29]	NM_172442
	Smad5, SMAD family member 5 [30]	NM_001164041
	Wnt2b, wingless-type MMTV integration site family, member 2B [31]	NM_009520
	Rptor, regulatory associated protein of MTOR complex 1 [32]	NM_028898
mmu-miR-361-3p	Map3k9, mitogen-activated protein kinase kinase kinase 9 [33]	NM_001174107
mmu-miR-713	Rag1, recombination activating gene 1 [34]	NM_009019
	Gprc5a, G protein-coupled receptor, family C, group 5, member A [35]	NM_181444
mmu-miR-706	Dusp3, dual specificity phosphatase 3 [36]	NM_028207
	Ncoa1, nuclear receptor coactivator 1[37]	NM_010881
	Nkiras1, NFKB inhibitor interacting Ras-like protein 1[38]	NM_023526
mmu-miR-703	ZnT4, solute carrier family 30 [39]	NM_011774
	Smad5, SMAD family member 5 [30]	NM_001164041
mmu-miR-574-3p	Tgfbr3, transforming growth factor, beta receptor III [40]	NM_011578
	Dicer1, dicer 1, ribonuclease type III [41]	NM_148948
	Pdk1, pyruvate dehydrogenase kinase, isoenzyme 1 [42]	NM_172665
mmu-miR-467b-3p	Runx2, runt-related protein 2 [43]	NM_001145920
	Pdgfra, platelet derived growth factor receptor alpha [44]	NM_001083316
	Mapk10, mitogen-activated protein kinase 10 [45]	NM_009158
mmu-miR-466i-5p	Atm, ataxia telangiectasia mutated [46]	NM_007499
	Rgs2, regulator of G-protein signaling 2 [47]	NM_009061
	Smo, smoothened, frizzled class receptor [48]	NM_176996
mmu-miR-466f-5p	Rcan1, regulator of calcineurin 1[49]	NM_001081549
	Clock, circadian locomotor output cycles kaput [50]	NM_007715
	Aff3, AF4/FMR2 family, member 3 [51]	NM_010678
mmu-miR-208a-3p	Il6ra, interleukin 6 receptor [52]	NM_010559
	Acvr1c, activin A receptor type 1C [40]	NM_001111030
	Sox6, SRY-Box 6 [40]	NM_011445
	Sox5, SRY-Box 5 [40]	NM_011444

For the target genes which are involved in bone formation, the references are provided

**Fig. 4:**
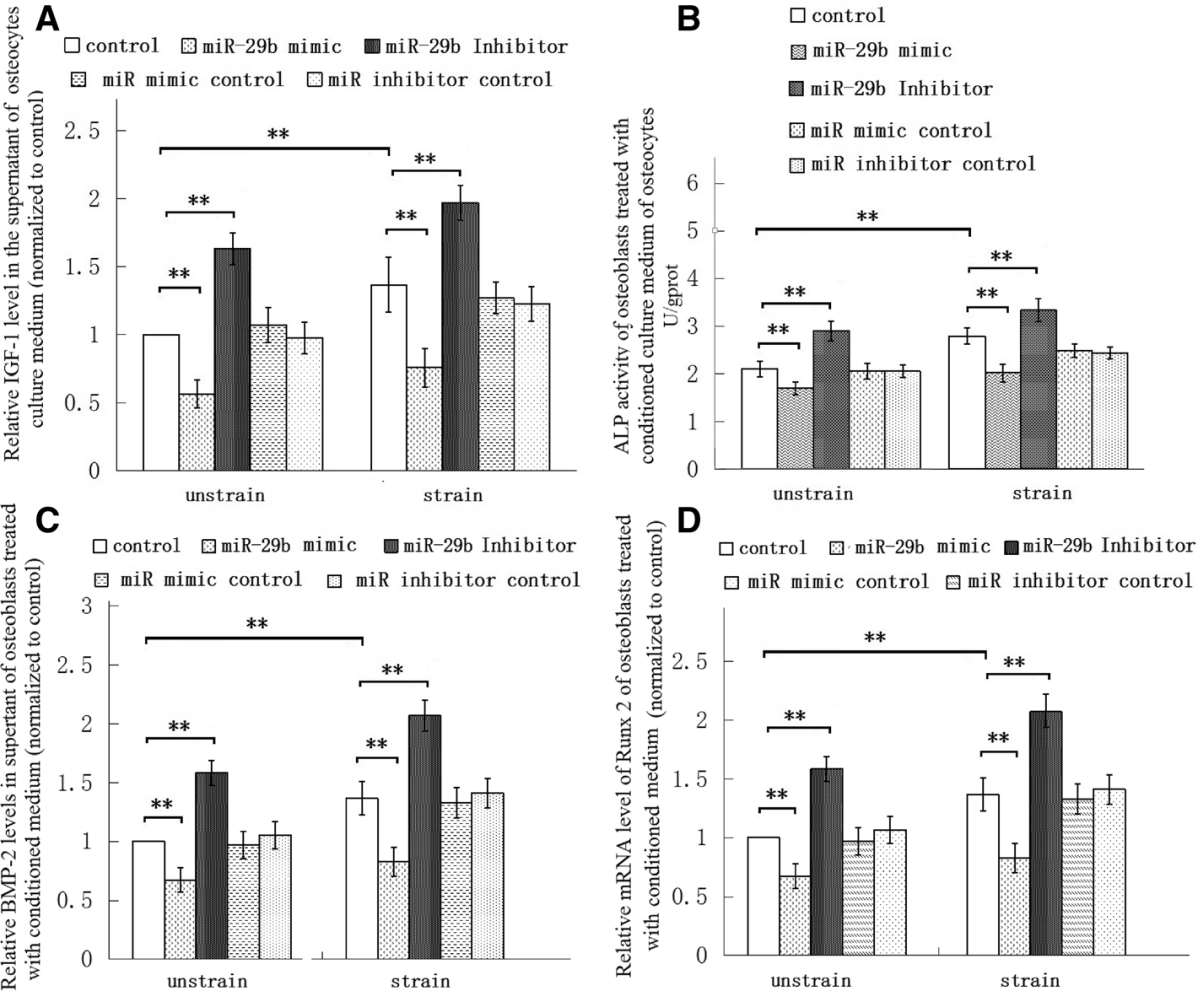
miR-29b-3p regulated IGF-1 secretion of MLO-Y4 osteocytes, and after the osteocytes were transfected with miR-29b-3p mimic or miR-29b-3p inhibitor, the osteocytes’ conditioned culture medium influenced osteoblastic differentiation of MC3T3-E1 cells. **a** miR-29b-3p mimic decreased IGF-1 secretion of osteocytes, and the miR inhibitor increased the IGF-1 secretion (*n* = 6). The conditioned medium of osteocytes treated with miR-29b-3p mimic reduced ALP activity of osteoblasts (**b**), BMP-2 protein (**c**) and Runx 2 mRNA (**d**) levels of osteoblasts (*n* = 6). In contrast, the conditioned medium of osteocytes treated with miR-29b-3p inhibitor enhanced ALP activity of osteoblasts (**b**), BMP-2 protein (**c**) and Runx 2 mRNA (**d**) levels of osteoblasts (*n* = 6). *P < 0.05 and **P < 0.01, compared with the unstrained control group, or between the indicated groups

Finally, we went on study to investigate the involvement of miR-29b-3p in osteocytes’ response to mechanical strain and the effect of the miR on osteoblast differentiation. We found that mechanical tensile strain down-regulated miR-29b-3p expression of MLO-Y4 cells, not MC3T3-E1 cells, and miR-29b-3p inhibited IGF-1 secretion of osteocytes (Fig. 3c). The miR-29b-3p mimic transfection resulted in over-expression of the miR in MLO-Y4 cells, which caused a reduction of IGF-1 levels in osteocytes’ culture supernatant, and miR-29b-3p inhibitor transfection, which resulted in low miR-29b-3p expression, and increased the IGF-1 secretion (Fig. 3b, Fig. 4a). In addition, the conditioned medium of MLO-Y4 cells transfected with miR-29b-3p mimic decreased ALP activity, and reduced expression of BMP-2 and Runx 2 in MC3T3-E1 cells (Fig. 4 b-d). In contrast, the conditioned medium of MLO-Y4 cells transfected with miR-29b-3p inhibitor increased ALP activity and enhanced expression of BMP-2 and Runx 2 in MC3T3-E1 cells (Fig. 4 b-d). These results indicated that miR-29b-3p regulated osteoblast differentiation via osteocyte secretion.

## Discussion

In this study, the results indicated that osteocytes responded to a mechanical tensile strain of 2500 με at 0.5 Hz for 8 h. Following osteocytes’ response to this mechanical loading, 10 mechanoresponsive miRs in MLO-Y4 osteocytes were identified, bioinformatics analysis revealed the possible target genes of these miRs, and 29 target genes were correlated with bone formation.

Among the mechanoresponsive miRs and their target genes, the relationship between miR-29b-3p (1 of 10 mechanoresponsive miRNAs) and its target gene, IGF-1, promoted us to find the regulatory relation of osteocytes to osteoblasts under the same mechanical stimulation. In this study, in MC3T3-E1 osteoblastic cells, miR-29b-3p was not involved in osteoblasts’ response to the mechanical strain, and the conditioned medium of the mechanically strained osteocytes promoted osteoblast differentiation of MC3T3-E1 cells. Additionally, the over-expression of miR-29b-3p inhibited osteoblast differentiation via reducing the IGF-1 level in the conditioned culture medium of MLO-Y4 osteocytes which was used to culture MC3T3-E1 cells. During the response to mechanical strain, miR-29b-3p had no direct effect on osteoblasts, and the miR regulated osteoblast differentiation via a mediator: osteocytes, because the mechanical strain up-regulated the cells’ miR-29b-3p, which reduced IGF-1 secretion of MLO-Y4 osteocytes.

Osteocytes in the lacunar-canalicular system of the bone are mainly mechanosensory cells in bone tissue. They transduce mechanical stimulation into biomechanical signals and regulate bone remodeling by regulating the activities of osteoblasts and osteoclasts [19, 20]. In this study, during the response to mechanical strain, miR-29b-3p had no direct effect on osteoblasts, and the miR regulated osteoblast differentiation via a mediator: osteocytes, because mechanical strain up-regulated the cells’ miR-29b-3p, which reduced IGF-1 secretion of MLO-Y4 osteocytes.

It has been reported that osteocytes are responsive to fluid shear stress, microgravity and fluid flow [21, 22]. The results of our study suggested that osteocytes could also respond to mechanical tensile strain and regulate osteoblast differentiation via miR-29b-3p regulating of IGF-1 secretion, which will shed some light on how osteocytes regulate osteogenesis.

## Conclusions

Osteocytes responded to a cyclic mechanical tensile strain of 2500 με at 0.5 Hz for 8 h, mechanoresponsive miRs were discovered in osteocytes, miR-29b-3p was responsive to the mechanical tensile strain and the miR regulated osteoblast differentiation via regulating IGF-1 secretion of osteocytes.

## Abbreviations

Bcl-2/Bax: B-cell lymphoma 2/Bax
BMP-2: Bone morphogenetic protein 2
COX2: Cyclooxygenase 2
ECL: Electrochemiluminescence
ELISA: Enzyme-linked immunosorbent assay
IGF-1: Insulin-like growth factor-1
miR: microRNA
NO: Nitric oxide
NOS: Nitric oxide synthase
OPG: Osteoprotegerin
PGE2: Prostaglandin E2
PVDF: Polyvinylidene fluoride membrane
RANKL: Receptor activator of NF-kB ligand
SDS: Sodium dodecyl sulfate
με: Microstrain
